# Anomaly detection via Gumbel Noise Score Matching

**DOI:** 10.3389/frai.2024.1441205

**Published:** 2024-09-24

**Authors:** Ahsan Mahmood, Junier Oliva, Martin Andreas Styner

**Affiliations:** Department of Computer Science, University of North Carolina at Chapel Hill, Chapel Hill, NC, United States

**Keywords:** anomaly, detection, categorical, unsupervised, tabular, score matching

## Abstract

We propose Gumbel Noise Score Matching (GNSM), a novel unsupervised method to detect anomalies in categorical data. GNSM accomplishes this by estimating the scores, i.e., the gradients of log likelihoods w.r.t. inputs, of continuously relaxed categorical distributions. We test our method on a suite of anomaly detection tabular datasets. GNSM achieves a consistently high performance across all experiments. We further demonstrate the flexibility of GNSM by applying it to image data where the model is tasked to detect poor segmentation predictions. Images ranked anomalous by GNSM show clear segmentation failures, with the anomaly scores strongly correlating with segmentation metrics computed on ground-truth. We outline the score matching training objective utilized by GNSM and provide an open-source implementation of our work.

## 1 Introduction

Anomaly detection on tabular data remains an unsolved problem (Pang et al., 2021b; Ruff et al., 2021; Aggarwal, 2017). Notably, there are few methods in this space that explicitly model categorical data types (Pang et al., 2021a). For instance, none of the methods tested in the recent comprehensive benchmark performed by Han et al. (2022) make explicit use of categorical information. After transforming the categorical variables into one-hot and binary encodings, existing methods proceed to treat them as distinct continuous variables. Furthermore, there is a dearth of unsupervised deep learning anomaly detection methods that excel on tabular datasets. For example, the otherwise exhaustive benchmark of Han et al. (2022) reports only two unsupervised deep learning models, DSVDD (Ruff et al., 2018) and DAGMM (Zong et al., 2018), in their analysis; with both models being outperformed by shallow unsupervised methods. Some reconstruction-based autoencoder approaches have been proposed (Hawkins et al., 2002) but they require optimization tricks such as adaptive sampling, pretraining, and ensembling to work effectively (Chen J. et al., 2017).

To fill this gap, we propose a novel unsupervised method to detect anomalies: Gumbel Noise Score Matching (GNSM). Our method estimates the scores of continuous relaxations of categorical variables. Our proposed method will naturally respect dependencies between feature indices of one-hot encoded covariates (instead of treating them as separate features), and yields a straightforward approach to model mixed continuous/discrete features through estimated scores.

Our main contributions are :

- Deriving an unsupervised training objective for learning the scores of categorical distributions.
- Demonstrating the capability of score matching for anomaly detection on categorical types in both tabular and image datasets.
- Providing a unified framework for modeling mixed data types via score matching.

To illustrate the significance of our last contribution, consider the Census dataset in our experiments (Section 5). We were able to compute the scores for both the continuous features [using standard denoising score matching (Vincent, 2011)] and the categorical features (using GNSM). Further still, our model is not limited to tabular data. As demonstrated in Section 6.2, GNSM can effectively detect anomalies in images (segmentation masks). This flexibility, paired with our simple loss objective, illustrates the practical viability of our method.

## 2 Background

Our work combines continuous relaxations for categorical data (Jang et al., 2017; Maddison et al., 2017) into the denoising score matching objective (Vincent, 2011). We will briefly expand on some background material to provide context.

### 2.1 Score matching

Let *x*∈ℝ^*D*^ be a sample observed from the probability distribution *p*(*x*), and $\tilde{x}\in {\mathbb{R}}^{D}$ represent the corrupted version of *x* under some noise distribution $q_{\sigma }\left(\tilde{x}|x\right)$, with a noise scale σ. Hyvärinen (2005) introduced score matching as a methodology to estimate the gradient of the log density with respect to the data (i.e., the score): ∇_*x*_log*p*(*x*). If we assume a noise distribution $q_{\sigma }\left(\tilde{x}|x\right)$ is available, it is possible to learn the scores for the perturbed data distribution $q_{\sigma }\left(\tilde{x}\right)≜\int q_{\sigma }\left(\tilde{x}|x\right)p\left(x\right)dx$. Vincent (2011) proved that that minimizing the Denoising Score Matching (DSM) objective in Equation 1 will train the score estimator *s*_θ_ to satisfy *s*_θ_(*x*) = ∇_*x*_log*q*_σ_(*x*).


(1) $$\begin{array}{l} J_{\text{DSM}}\left(\theta \right)={𝔼}_{q_{\sigma }}\left[||s_{\theta }\left(x\right)-\nabla_{\tilde{x}}\log q_{\sigma }\left(\tilde{x}|x\right)|{|}^{2}\right] \end{array}$$

Song and Ermon (2019) introduced Noise Conditioned Score Networks (NCSN) and expanded the DSM objective in Equation 1 to include multiple noise distributions of increasing noise levels.


(2) $$\begin{array}{l} J_{\text{NCSN}}\left(\theta \right)=\overset{L}{\underset{i=1}{\sum }}{𝔼}_{q_{\sigma_{i}}}\left[||s_{\theta }\left(x,\sigma_{i}\right)-\nabla_{\tilde{x}}\log q_{\sigma_{i}}\left(\tilde{x}|x\right)|{|}^{2}\right] \end{array}$$

The authors' main insight was to use the same model for all noise levels. They parameterized the network to accept noise scales as conditioning information. NCSNs were successful in generating images and have been shown to have close ties to generative diffusion models (Song and Ermon, 2019).

### 2.2 Connecting score matching to anomaly detection

While (Song and Ermon, 2019) demonstrated the generative capabilities of NCSNs, Mahmood et al. (2021) outlined how these networks can be repurposed for outlier detection. Their methodology, Multiscale Score Matching Analysis (MSMA), incorporates noisy score estimators to separate in- and out-of-distribution (OOD) points. Recall that a score is the gradient of the likelihood. A typical point, residing in a space of high probability density will need to take a small gradient step in order to improve its likelihood. Conversely, a point further away from the typical region (an outlier) will need to take a comparatively larger gradient step toward the high density region. When we have multiple noisy score estimates, it is difficult to know apriori which noise scale accurately represents the gradient of the outliers. However, Mahmood et al. (2021), showed that learning the typical space of score-norms for all noise levels is sufficient to identify anomalies.

Concretely, assume we have a score estimator that is trained on *L* noise levels and a set of inlier samples *X*_IN_. Computing the inlier score estimates for all noise levels and taking the L2-norms across the input dimensions results in an *L*-dimensional feature vector for each input sample: $\left[||s\left(X_{\text{IN}},\sigma_{1}\right)|{|}_{2}^{2},...,||s\left(X_{\text{IN}},\sigma_{L}\right)|{|}_{2}^{2}\right]$. Mahmood et al. (2021) argue that inliers tend to concentrate in this multiscale score-norm embedding space. It follows that one could train an auxiliary model (such as a clustering model or a density estimator) to learn this score-norm space of inliers. At test time, the output of the auxiliary model (e.g., likelihoods in the case of density estimators) is used as an anomaly score. Results in Mahmood et al. (2021) show MSMA to be effective at identifying OOD samples in image datasets (e.g., CIFAR-10 as inliers and SVHN as OOD).

### 2.3 Continuous relaxation to categorical data

Gradients of log likelihoods are ill-defined for categorical inputs. In order to compute the score of categorical data, we propose to adopt a continuous relaxation for discrete random variables co-discovered by Jang et al. (2017) and Maddison et al. (2017). These relaxations build on the Gumbel-Max trick to sample from a categorical distribution (Maddison et al., 2014). The procedure (often referred to as the Gumbel-Softmax) works by adding Gumbel noise (Gumbel, 1954) to the (log) probabilities and then passing the resulting vector through a softmax to retrieve a sharpened probability distribution over the categorical outcomes. Of particular interest to us, Gumbel-Softmax incorporates a temperature parameter (λ in Equation 3) to control the sharpening of the resulting probabilities. we argue that this temperature can also be interpreted as a noise parameter, by virtue of it increasing the entropy of the post-softmax probabilities. we will make use of this intuition to combine continuous relaxations with denoising score matching.

Note that for our analysis in Section 3, we will be utilizing the formulation of Maddison et al. (2017) i.e. concrete random variables. In particular, we will be using a variant of the Concrete Distribution called ExpConcrete introduced by the same authors, shown in Equation 3. Given unnormalized probabilities (logits) of a *K*-dimensional variable α∈(0, ∞)^*K*^, Gumbel i.i.d samples *G*_*k*_, and a smoothing factor λ∈(0, ∞), we can construct an ExpConcrete random variable *X*∈ℝ^*K*^ such that exp(*X*)~Concrete(α, λ):


(3) $$\begin{array}{l} X_{k}=\frac{\log\alpha_{k}+G_{k}}{\lambda }-\log\overset{K}{\underset{i=1}{\sum }}\exp\left\{\frac{\log\alpha_{i}+G_{i}}{\lambda }\right\} \end{array}$$

As λ → 0, the computation approaches an argmax, while large values of λ will push the random variable toward a uniform distribution. The main purpose of preferring the ExpConcrete Distribution over the Concrete Distribution is numerical stability, as the former is defined in the log domain.

Conveniently, Maddison et al. (2017) derived the log-density of an ExpConcrete random variable, which we will be using going forward. Let *x*∈ℝ^*K*^ such that the $\log\overset{K}{\underset{i=1}{\sum }}\exp\left\{x_{i}\right\}=0$. The log-density of an ExpConcrete(α, λ) distribution can be computed as:


(4) $$\begin{array}{l} \;\log p_{\alpha ,\lambda }(x)=\log((K-1)!)+(K-1)\log\lambda \\ +\left(\sum_{k=1}^{K}\log\alpha_{k}-\lambda x_{k}\right)-K\log\sum_{k=1}^{K}e^{\left(\log\alpha_{k}-\lambda x_{k}\right)} \end{array}$$

## 3 Score matching with categorical variables

In this section we will develop the ideas behind our loss objective. Firstly, note that the proof of the denoising score matching objective in Equation 1, introduced by Vincent (2011), holds true for any *q*_σ_, provided that $\log q_{\sigma }\left(\tilde{x}|x\right)$ is differentiable. Recall that *q*_σ_ plays the role of a noise distribution. While most denoising score matching models incorporate Gaussian perturbation (Song and Ermon, 2019; Song et al., 2021; Vincent, 2011), we emphasize that *any* noise distribution may be used during training.

### 3.1 ExpConcrete(α,λ) as a noise distribution

Following the reasoning above and the temperature parameter (λ) available in Equation 3, we propose to repurpose the Concrete distribution to add “noise” to our continuous relaxations of the categorical variables. Increasing λ will allow us to corrupt the input *x* by scaling the logits and smoothing out the categorical probabilities. Therefore, in GNSM, the (Exp)Concrete Distribution acts both as the relaxation mechanism *and* the noise distribution.

Let **x**∈{0, 1}^*K*^ be a one-hot encoding representing *K* outcomes and *x*~ExpConcrete(α = **x**, λ)∈ℝ^*K*^ be the continuously relaxed approximation of the one-hot vector **x**. We set α to be the logits of **x**. As **x** will be a one-hot encoding, it does not strictly satisfy the requirement α∈(0, ∞)^*K*^. This can be circumvented by adding a small delta to the vectors to avoid zero values i.e. α = **x**+δ. While it is possible to apply any transformation to convert **x** to unnormalized probabilities, we opted to use the clamped one-hot encodings for simplicity.

Let $\tilde{x}\sim \text{ExpConcrete}\left(\alpha =\text{x},\lambda >>0\right)\in {\mathbb{R}}^{K}$ represents the noisy version of **x** with a smoothing factor λ>>0 being used to excessively smooth the probabilities of *x*. We compute the log-density of the noise distribution as:


(5) $$\begin{array}{lll} \log q_{\sigma }\left(\tilde{x}|x\right) & = & \log p_{\alpha ,\lambda }\left(\tilde{x}|x\right) \end{array}$$

(6) $$\begin{array}{ll} = & \log p_{\lambda }\left(\tilde{x};\alpha =\text{x}\right) \end{array}$$

Here, the location parameter is that of the unperturbed input (similar to how one would use a Gaussian kernel), and λ is a known hyperparameter.

### 3.2 Score of ExpConcrete distribution

To plug ExpConcrete into the DSM objective (Equation 1), we first need to derive the score for the ExpConcrete distribution i.e. take the gradient of the log-density with respect to the data.

Recall the log-density of the ExpConcrete(α, λ) in Equation 4. Since the first two terms for log*p*_α, λ_(*x*) in Equation 4 are independent of *x*, we can ignore them and focus on the latter:


(7) $$\begin{array}{l} \;\log p_{\alpha ,\lambda }(x)\\ =\log((K-1)!)+(K-1)\log\lambda \;+\left(\sum_{k=1}^{K}\log\alpha_{k}-\lambda x_{k}\right)\\ \;-K\log\sum_{k=1}^{K}e^{\left(\log\alpha_{k}-\lambda x_{k}\right)} \end{array}$$

(8) $$\begin{array}{l} \nabla_{x_{j}}\log p_{\alpha ,\lambda }(x)=\nabla_{x_{j}}\left(\sum_{k=1}^{K}\log\alpha_{k}-\lambda x_{k}\right)\\ \;-\nabla_{x_{j}}\left(K\log\sum_{k=1}^{K}\exp\left\{\log\alpha_{k}-\lambda x_{k}\right\}\right) \end{array}$$

(9) $$\begin{array}{l} \;=\nabla_{x_{j}}\left(-\sum_{k=1}^{K}\lambda x_{k}\right)\\ -K\left(\nabla_{x_{j}}\log\sum_{k=1}^{K}\exp\left\{\log\alpha_{k}-\lambda x_{k}\right\}\right) \end{array}$$

(10) $$\begin{array}{l} =-\lambda -K\frac{\nabla_{x_{j}}\left(\sum_{k=1}^{K}\exp\left\{\log\alpha_{k}-\lambda x_{k}\right\}\right)}{\sum_{k=1}^{K}\exp\left\{\log\alpha_{k}-\lambda x_{k}\right\}} \end{array}$$

(11) $$\begin{array}{l} =-\lambda -K\frac{\exp\left\{\log\alpha_{j}-\lambda x_{j}\right\}\nabla_{x_{j}}\left(\log\alpha_{j}-\lambda x_{j}\right)}{\sum_{k=1}^{K}\exp\left\{\log\alpha_{k}-\lambda x_{k}\right\}} \end{array}$$

(12) $$\begin{array}{l} =-\lambda -K\frac{\exp\left\{\log\alpha_{j}-\lambda x_{j}\right\}\left(-\lambda \right)}{\sum_{k=1}^{K}\exp\left\{\log\alpha_{k}-\lambda x_{k}\right\}} \end{array}$$

(13) $$\begin{array}{l} =-\lambda +\lambda K\;\frac{\exp\left\{\log\alpha_{j}-\lambda x_{j}\right\}}{\sum_{k=1}^{K}\exp\left\{\log\alpha_{k}-\lambda x_{k}\right\}} \end{array}$$

Note how the last equation can be rewritten as:


(14) $$\begin{array}{l} \nabla_{x_{j}}\log p_{\alpha ,\lambda }\left(x\right)=-\lambda +\lambda K\;\sigma {\left(\log\alpha -\lambda x\right)}_{j} \end{array}$$

where $\sigma {\left(\text{z}\right)}_{i}=\frac{e^{z_{i}}}{\overset{K}{\underset{k=1}{\sum }}e^{z_{k}}}$ is the softmax function.

### 3.3 Gumbel-Noise Score Matching Objective

Equation 14 represents the score function of the ExpConcrete distribution i.e. the gradient of the log-density with respect to the data. We can now combine the ideas from Denoising Score Matching and Concrete random variables. Combining Equations 1, 5, 14, one obtains


$$\begin{array}{l} J(\theta )=E_{q_{\sigma }}\left[||s_{\theta }(x)-\nabla_{\tilde{x}}\log q_{\sigma }(\tilde{x}|x)|{|}^{2}\right]\\ \;=E_{p_{\lambda }}\left[||s_{\theta }(\tilde{x})-\nabla_{\tilde{x}}\log p_{\alpha ,\lambda }(\tilde{x}|x)|{|}^{2}\right]\\ \;=E_{p_{\lambda }}\left[||s_{\theta }(\tilde{x})-\nabla_{\tilde{x}}\log p_{\lambda }(\tilde{x};\alpha =x)|{|}^{2}\right]\\ \;=E_{p_{\lambda }}\left[||s_{\theta }(\tilde{x})-(-\lambda +\lambda K\;\sigma (\log x-\lambda \tilde{x}))|{|}^{2}\right]\\ \;=E_{p_{\lambda }}\left[||s_{\theta }(\tilde{x})-\lambda K\;\sigma (\log x-\lambda \tilde{x}))+\lambda |{|}^{2}\right]\\ \;=E_{p_{\lambda }}\left[||s_{\theta }(\tilde{x})-\lambda K\;\sigma (\epsilon )+\lambda |{|}^{2}\right] \end{array}$$

Here $\epsilon =\log\text{x}-\lambda \tilde{x}$ and can be loosely interpreted as the “logit noise” as it is the difference between the original logit probabilities and the perturbed vector. This formulation is analogous to the simplification utilized by Song et al. (2021) and Ho et al. (2020). It allows us to train the model to estimate the noise directly as the other variables are known constants. Assume a network ϵ_θ_, that takes the input $\tilde{x}$. Following Equation 14, we parameterize a score network as $s_{\theta }{\left(\tilde{x}\right)}_{j}=-\lambda +\lambda K\;\sigma {\left(\epsilon_{\theta }\left(\tilde{x}\right)\right)}_{j}$. We train the network ϵ_θ_ to estimate the noise values ϵ by the objective below.


(15) $$\begin{array}{l} J\left(\theta \right)={𝔼}_{p_{\lambda }}\left[\lambda^{2}K^{2}||\left(\sigma \left(\epsilon_{\theta }\left(\tilde{x}\right)\right)-\sigma \left(\epsilon \right)\right)|{|}^{2}\right] \end{array}$$

Following Song and Ermon (2019), we can modify our loss to train a NCSN with *L* noise levels i.e. $\lambda \in {\left\{\lambda_{i}\right\}}_{i=1}^{L}$:


(16) $$\begin{array}{l} J\left(\theta \right)=\sum_{i=0}^{L}\lambda_{i}^{2}K^{2}\;{𝔼}_{\text{x}\sim p_{\text{data}}}{𝔼}_{\tilde{x}\sim p_{\lambda_{i}}}\left[||\sigma \left(\epsilon_{\theta }\left(\tilde{x},\lambda_{i}\right)\right)-\sigma \left(\epsilon \right)|{|}^{2}\right] \end{array}$$

Note that our network is now additionally conditioned on the noise level λ. Finally, our loss objective can be extended to incorporate data with multiple categorical features. For *D* categories we have:


(17) $$\begin{array}{l} \;J_{GNSM}(\theta )\\ =\sum_{d=0}^{D}\sum_{i=0}^{L}\lambda_{i}^{2}K_{d}^{2}\;E_{x_{d}\sim p_{\text{data}}}E_{{\tilde{x}}_{d}\sim p_{\lambda_{i}}}\left[||\sigma (\epsilon_{\theta }({\tilde{x}}_{d},\lambda_{i}))-\sigma (\epsilon )|{|}^{2}\right] \end{array}$$

Here, *K*_*d*_ represents the number of outcomes per category, *x*_*d*_ represents the one-hot vector of length *K*_*d*_, and ${\tilde{x}}_{d}$ is the continuous, noisy representation of *x*_*d*_ obtained after a Concrete (Gumbel-Softmax) transform.

### 3.4 A note on optimizing the GNSM objective in practice

Observing the loss in Equation 17, we see that we are minimizing the difference between two distributions as both inner terms pass through a softmax function. This insight led us to postulate that that one could substitute the mean squared error loss (MSE) for a metric more apt for matching distributions. We therefore ran experiments using the KL divergence objective as shown in Equation 18. This objective showed faster convergence than MSE. Admittedly, this result is only empirical. It may be possible to gain similar improvements in convergence for the MSE by properly tuning the optimization hyperparameters such as the learning rate.


(18) $$\begin{array}{l} \;J_{GNSM}(\theta )\\ =\sum_{d=0}^{D}\sum_{i=0}^{L}\lambda_{i}^{2}K_{d}^{2}\;E_{x\sim p_{\text{data}}}E_{\tilde{x}\sim p_{\lambda_{i}}}\left[D_{\text{KL}}(\sigma (\epsilon )∥\sigma (\epsilon_{\theta }({\tilde{x}}_{d}))\right]\;\; \end{array}$$

### 3.5 Anomaly detection via GNSM-based MSMA

Once a network is trained with the denoising objective in Equation 17, we can plug the scores into MSMA to identify anomalies. For a given point *x*, we compute the score estimates for all noise perturbation levels. The resulting vector represents the *L*-dimensional multiscale embedding space:


(19) $$\begin{array}{l} \eta \left(x\right)=\left(||s_{\theta }\left(x,\lambda_{1}\right)|{|}_{2}^{2},...,||s_{\theta }\left(x,\lambda_{L}\right)|{|}_{2}^{2}\right) \end{array}$$

where *s*_θ_(*x*, λ_*i*_) is the noise conditioned score network estimating ∇_*x*_log*p*_λ_*i*__(*x*). Following the mechanism laid out by Mahmood et al. (2021), our goal is to learn “areas of *concentration*” of the inlier data in the *L*-dimensional embedding space (η(*x*), for *x*~*p*). Concretely, we train a Gaussian Mixture Model (GMM) on η(*X*_IN_), where *X*_IN_ represents the set of inliers. At inference time, we first use the score network to compute the score-embedding space η(*x*) for the test samples and then compute the likelihoods of the scores via the trained GMM. The negative of this likelihood is then assumed as the anomaly score for the test samples.

## 4 Related works

Unsupervised anomaly detection has been tackled by a myriad of methods (Pang et al., 2021b; Ruff et al., 2021), with varying success (Han et al., 2022). For the purposes of this work, we primarily focus on unsupervised anomaly detection algorithms that have been successfully applied to tabular data. Every algorithm employs its own assumptions and principles about normality (Aggarwal, 2017). These principles can be elucidated into three broad detection methodologies based on classification, distance and density.

Classification-based methods employ a one-class objective, which does not need labeled samples. For example, One-Class Support Vector Machines (OC-SVMs) (Chen et al., 2001) try to find the tightest hyperplane around the dataset, while Deep Support Vector Data Descriptors (DSVDD) (Ruff et al., 2018) will compute the minimal hypersphere that encloses the data. Both methods assume that inliers will fall under the margins, and consequently use the distance to the margin boundaries as a score of outlierness.

Distance-based methods assume that outliers will be far away from neighborhoods of inliers. For example, k-Nearest Neighbors (Peterson, 2009) will use the distance to the k-th nearest inlier point as a score of anomaly. Isolation Forests (Liu et al., 2008) implicitly use this assumption by computing the number of partitions required to isolate a point. Samples that are far away from their neighbors will thus be isolated with fewer partitions and be labeled as anomalies.

Lastly, density-based models assume that anomalies are located in low-density regions in the input space. The principle objective is then to learn the density function representative of the typical (training) data. A trained model will be used to assign probabilities to test samples, with low probabilities signifying anomalies. Examples include Gaussian Mixture Models (GMMs) (Reynolds et al., 2009) and their deep learning counter part, Deep Autoencoding Gaussian Mixture Models (DAGMM) (Zong et al., 2018). Both models estimate the parameters for a mixture of Gaussians, which are then used to assign likelihoods at inference time. ECOD (Li et al., 2022) uses a different notion of density and estimates the cumulative distribution function (CDF) for each feature in the data. It then uses the tail probabilities from each learned CDF to designate samples as anomalous.

There are also many methods built specifically for anomaly detection in images such as Schlegl et al. (2019), Bergmann et al. (2020), and Defard et al. (2021). However, they have yet to be successfully applied to tabular data and it is uncertain how to extend them to categorical data types. Conversely, some methods have been built to address *only* categorical data types such as Akoglu et al. (2012) (compression-based) and Pang et al. (2016, 2021a) (frequency-based). Unfortunately, it is difficult to find open-source implementations of these models. It is also non-obvious how to extend them to mixed continuous/discrete features. Our method on the other hand, can handle mixed data types by using the appropriate score matching objective for continuous and categorical features.

Finally, we emphasize that our research introduces a streamlined approach to estimate scores for categorical data using denoising score matching. Recently, Sun et al. (2023) proposed a ratio matching objective, which may be viewed as a discrete analog to score matching with continuous variables. However, this method mandates the parameterization of conditional densities, necessitating a crafted architecture to mask specific input segments. In contrast, our method sidesteps such complexities, and can fit into any established score matching framework. For example, our method is compatible with alternative (non-denoising) score matching objectives such as sliced-score matching (Song et al., 2020), or the implicit score matching objective originally proposed by Hyvärinen (2005).

There is also a link between score matching and diffusion models as established by Song et al. (2021). Indeed, recent works such as Austin et al. (2021) and Hoogeboom et al. (2021) model categorical distributions through a diffusion process. However, it is important to note that these generative models eschew the estimation of the score function *s*(*x*) = ∇_*x*_log*p*(*x*). Instead, they incorporate the Markov chain interpretation of diffusion models, and directly predict the parameters for transition kernels. As a consequence, these models are not directly suitable for a spectrum of score-based applications, such as out-of-distribution (OOD) detection as explored by Mahmood et al. (2021), or hypothesis testing as introduced by Wu et al. (2022). It is plausible that forthcoming research will unveil further applications of score functions, wherein our methodology stands ready to extend these findings to categorical data.

## 5 Experiments

We designed two experiments to evaluate our methodology: a benchmark on tabular data and a vision-based case study. The tabular benchmark will quantitatively assess the performance of GNSM compared to baselines. The case study will demonstrate a real world use case of detecting anomalous segmentation masks.

### 5.1 Tabular benchmark

We created an experimental testbed with categorical anomaly detection datasets sourced from a publicly available curated repository.^1^ Table 1 describes the public datasets used in our experiments. Note that for our method, we need to know the number of outcomes for each category, to appropriately compute the softmax over the dimensions. This prevents us from using preprocessed datasets such as those made available by Han et al. (2022). It is also why we could not use all the datasets in the curated repository, as some had been pre-binarized.

**Table 1:** Statistics of public benchmark datasets.

**Dataset**	**# Samples**	**# Anomalies**	**# Features**
Bank	36,548	4,640	53
Census	280,717	18,568	396 (+5 cont.)
Chess	28,029	27	40
CMC	1,444	29	25
Probe	60,593	4,166	67
Solar	1,023	43	41
U2R	60,593	228	40
Nursery	4,648	328	26

All datasets other than Census are categorical only.

We first split the datasets into inliers and outliers. Next, we divided the inliers into an 80/10/10 split for train, validation, and test respectively. The validation set is used for early stopping and the checkpoint with the best validation loss is used for inference. The test set is combined with the outliers and used for assessing performance. The categorical features were first converted to one-hot vectors and then passed through a log transform to retrieve logits. We used Standard normalization to normalize any continuous features (only relevant for Census). We compute results over five runs with different seeds.

We chose four methods to represent baseline performance in lieu of a comprehensive analysis with multiple methods. We were inspired to go this route due to the thorough results reported by ADBench (Han et al., 2022). As the authors describe, no one method outperforms the rest. We picked two representatives for shallow unsupervised methods: Isolation Forests and ECOD. We picked these as they consistently give good performance across different datasets and require little to no hyperparameter tuning. There are much fewer options for unsupervised deep learning methods that have been shown to work on tabular datasets. We chose two models that are popular in this field: DAGMM and DSVDD. Note that these were the only unsupervised deep learning models reported by Han et al. (2022).

For our score network, we used a ResNet-like architecture inspired by Gorishniy et al. (2021). We replaced BatchNorm layers with LayerNorm and set Dropout to zero. The dimensions of the Linear layers in each block were set to 1,024. All activations were set to GELU (Hendrycks and Gimpel, 2016) except for the final layer, which was set to LeakyReLU. The number of residual blocks was set to 20. To condition the model on the noise scales, we added a noise embedding layer similar to those used in diffusion models (Song et al., 2021). We used the same architecture across all datasets.

Our noise parameter λ is a geometric sequence from λ = 2 to λ = 20. Early testing showed that the models gave numerical issues for values lower than 2. For the upper-limit (i.e. the largest noise scale) we chose 20 as it works well to smooth out the probabilities to uniform across all datasets. We set the number of noise scales (*L*) to 20. We compute the score norms on the inliers (train+val) according to Equation 19 and train a GMM on the resulting features. The negative likelihoods computed from the GMM are the final outputs of our method.

Extensive architectural details are available in the Appendix. We also provide our code at the categorical-dsm code repository.^2^

### 5.2 Case study: detecting segmentation failures

Consider the scenario where a user has deployed a (trained) segmentation model and wishes to detect when the model fails to produce adequate segmentations during inference. This case-study will explore how we can use a GNSM to rank (image, segmentation) pairs, where the segmentations are predictions from a deep learning model. Effectively, we aim to show that a GNSM network can act as an uncertainty estimator for the outputs of a pre-trained segmentation model.

Concretely, the GNSM model is first trained to learn the distribution of ground truth (image, segmentation) pairs. At test time, our model will score the predicitons of a pretrained segmentation model. Our hypothesis is that our method will correctly detect failure cases i.e. poor segmentations should be ranked higher on the anomaly scale.

While there are many ways to qualitatively define a failure, we will be using popular segmentation metrics (with respect to ground truth masks) as a proxy for performance. We posit that a useful anomaly score should correlate meaningfully with the ground truth segmentation accuracies.

We compare the anomaly scores against three common segmentation metrics: the Dice similarity coefficient (Dice), the mean surface distance (MSD), and the 95-th percentile Hausdorff distance (95-HD). We chose Dice as it is a popular segmentation metric that measures the overlap between the predicted masks and the ground truth. However, as Dice scores may overestimate performance, it is recommended to additionally report distance based metrics (Valentini et al., 2014; Taha and Hanbury, 2015). These metrics compute the distance between the surfaces of the predictions and ground truth masks.

We train a convolutional score network on the train-set of the Pascal-VOC segmentation dataset (Everingham et al., 2010). The input to our model is a pair of images and the one-hot segmentation masks. The model predicts the scores for the segmentation masks only. We chose to use paired data rather than segmentations alone as we want the model to learn whether a segmentation is appropriate for the *given* image.

As our test subject, we retrieved a pretrained DeepLabV3 MobileNet (V3 Large) segmentation model (Chen L.-C. et al., 2017) from the publicly available PyTorch implementation.^3^ This model was trained on a subset of the COCO dataset (Lin et al., 2014), using only the 20 categories that are present in the Pascal VOC dataset. We used the validation set of Pascal VOC as our test set.

We compare the performance of our method to a convolutional DSVDD. While there may exist specialized segmentation uncertainty estimators, we argue that an unsupervised model provides a more apt comparison. It is reasonable to postulate that both our model and DSVDD could be improved by additionally incorporating segmentation-specific objectives into the training, but that remains outside the scope of this study.

For our score matching network, we adopted the NCSN++ model used by Song et al. (2021). The only significant change was in the input/output layers as we are predicting scores over one-hot segmentation masks. For DSVDD, we used the implementation of the original authors (Ruff et al., 2018). To keep a fair comparison, we modified the code to use a modern architecture as the backbone [specifically EfficentNetV2 (Tan and Le, 2021)] and kept the number of parameters similar to our model. Both models were trained to convergence and the best checkpoints (tested over a validation split of the train-set) were used for the analysis.

## 6 Results

### 6.1 Performance on tabular benchmark

We report the Average Precision error (AP) which can also be interpreted as the Area Under the Precision Recall curve (AUPR). Average precision computes the mean precision over all possible detection thresholds. We chose to highlight AP over AUROC as it is a more apt measure for detecting anomalies, where we often have unbalanced classes. Additionally, precision measures the positive predictive value of a classification i.e. the true positive rate. This is a particularly informative measure for anomaly detection algorithms where we are preferentially interested in the performance over one class (outliers) than the other (inliers). We would also like to note that our anomaly ratios in the test set do not correspond with the true anomaly ratio in the original dataset. This is due to our data splitting scheme where our test set is effectively only 10% the size of inliers.

Table 2 shows that our approach performs better or on par with baselines. GNSM achieves significant performance improvements over baselines for Census, Probe and U2R, respectively achieving a 6.61%, 2.09%, and 11.18% improvement over the next best method.

**Table 2:** Average precision across multiple datasets.

**Dataset**	**Ano ratio**	**IForests**	**ECOD**	**DAGMM**	**DSVDD**	**GNSM (ours)**
Bank	0.56	63.24 ± 1.74	**66.52 ± 0.57**	57.62 ± 3.36	58.50 ± 5.30	65.58 ± 3.45
Census	0.40	40.64 ± 2.07	40.96 ± 0.15	32.90 ± 5.00	41.18 ± 3.44	**47.79 ± 2.29**
Chess	0.01	**2.31 ± 1.36**	1.43 ± 0.05	1.08 ± 0.44	1.47 ± 0.54	1.60 ± 0.68
CMC	0.17	22.72 ± 1.57	23.79 ± 1.75	24.99 ± 5.75	21.99 ± 6.15	**25.87 ± 9.93**
Probe	0.41	92.95 ± 2.28	95.39 ± 0.38	66.40 ± 9.43	89.16 ± 8.40	**97.48 ± 0.62**
Solar	0.30	67.99 ± 3.48	**72.23 ± 0.91**	50.84 ± 5.19	51.21 ± 3.94	69.28 ± 1.96
U2R	0.04	52.74 ± 12.88	67.84 ± 1.39	10.06 ± 6.47	71.17 ± 24.65	**82.35 ± 5.45**
Nursery	0.43	46.51 ± 6.52	**100.00 ± 0.00**	48.33 ± 8.64	**100.00 ± 0.00**	**100.00 ± 0.01**
Average	-	48.64 ± 3.99	58.52 ± 0.74	36.52 ± 5.53	54.33 ± 7.49	**61.24 ± 3.05**

Higher is better. Each experiment was repeated with five different seeds and we report the mean and standard deviations across seeds. IForest and ECOD represent shallow models, while DAGMM and DSVDD represent deep learning models. Ano ratio refers to the ratio of anomalies in the test set. Bold values indicate the highest average precision across all methods per dataset.

Results for CMC and Bank are less straightforward to interpret as the differences in the models are not statistically significant, made apparent by the large overlap in the standard deviations. This is especially true for deep learning models which have to be optimized via gradient descent. On Solar, ECOD outperforms the rest by a significant margin. However, between deep learning models, GNSM performs notably better. Note that Solar is the smallest dataset in our testing, with less than 800 training samples. Lastly, every model struggled with Chess, quite possibly due to the exceptionally small anomaly ratio. While Isolation Forests achieves the highest mean, it is uncertain whether the win is statistically significant. One could easily opt in favor of the other methods for this dataset as they achieve more consistent results. Again, between deep learning models, GNSM performs better.

Overall, we observed that the shallow models give more stable and consistent results, with ECOD having the smallest standard deviations on average. Additionally, we note that the reported tabular datasets prove difficult for all algorithms. This behavior is prevalent in the field of unsupervised anomaly detection methods, where models exhibit a large variance in accuracy across datasets (Han et al., 2022). As such, no one method definitively outperforms the rest; an outcome that coincides with previous findings of Han et al. (2022); Pang et al. (2021b), and Ruff et al. (2021). In this context, we emphasize that GNSM consistently ranks high across all datasets we tested. In contrast, each of the competing methods were the top performer in only one of the datasets in Table 2, and significantly underperformed in others. Averaged over all datasets, GNSM performed best. This is empirical confirmation that GNSM is a consistent contender in the suite of available algorithms for practitioners looking to detect anomalies in unlabeled data domains.

### 6.2 Detecting segmentation failures

We computed the anomaly scores from both GNSM and DSVDD and ranked the images from most to least anomalous. Next, we took the top *K* = 50 images (out of 1449) and computed the Pearson correlation coefficients between the ground truth segmentation metrics and the anomaly scores. We chose the worst ranked images for our analysis as we are interested in the efficacy of these scores for identifying segmentation failures as opposed to assessing the quality of successful segmentations.

**Figure 1:**
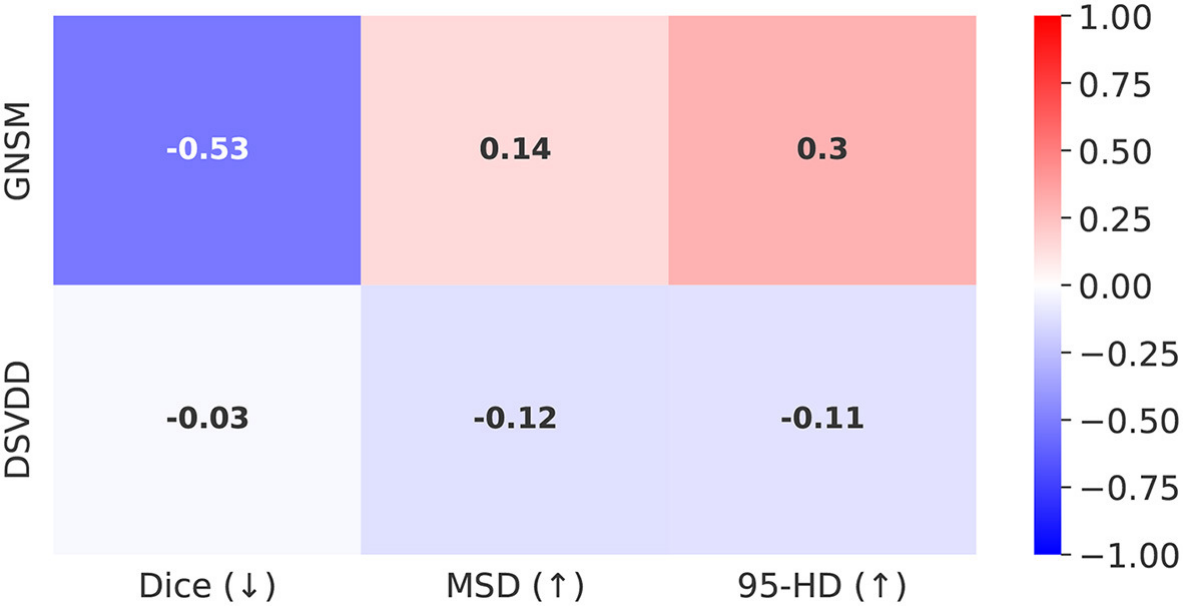
Correlations with segmentation metrics for Top-*K* = 50 anomaly scores retrieved from GNSM and Deep SVDD. The arrows next to the metric denote the expected correlation direction. The magnitude of the correlations reflects how well the anomaly scores capture segmentation errors.

Figure 1 shows the correlations between the ground truth segmentation metrics and the anomaly scores from GNSM and DSVDD. Recall that Dice is a similarity metric while MSD and 95-HD are both distance-based metrics. Therefore, we initially hypothesized that a good anomaly score should correlate negatively with Dice and positively with the distances. Our results show that GNSM correlates strongly in the direction expected. DSVDD on the other hand achieved a poor correlation with Dice and inverse correlations with the distance based metrics.

**Figure 2:**
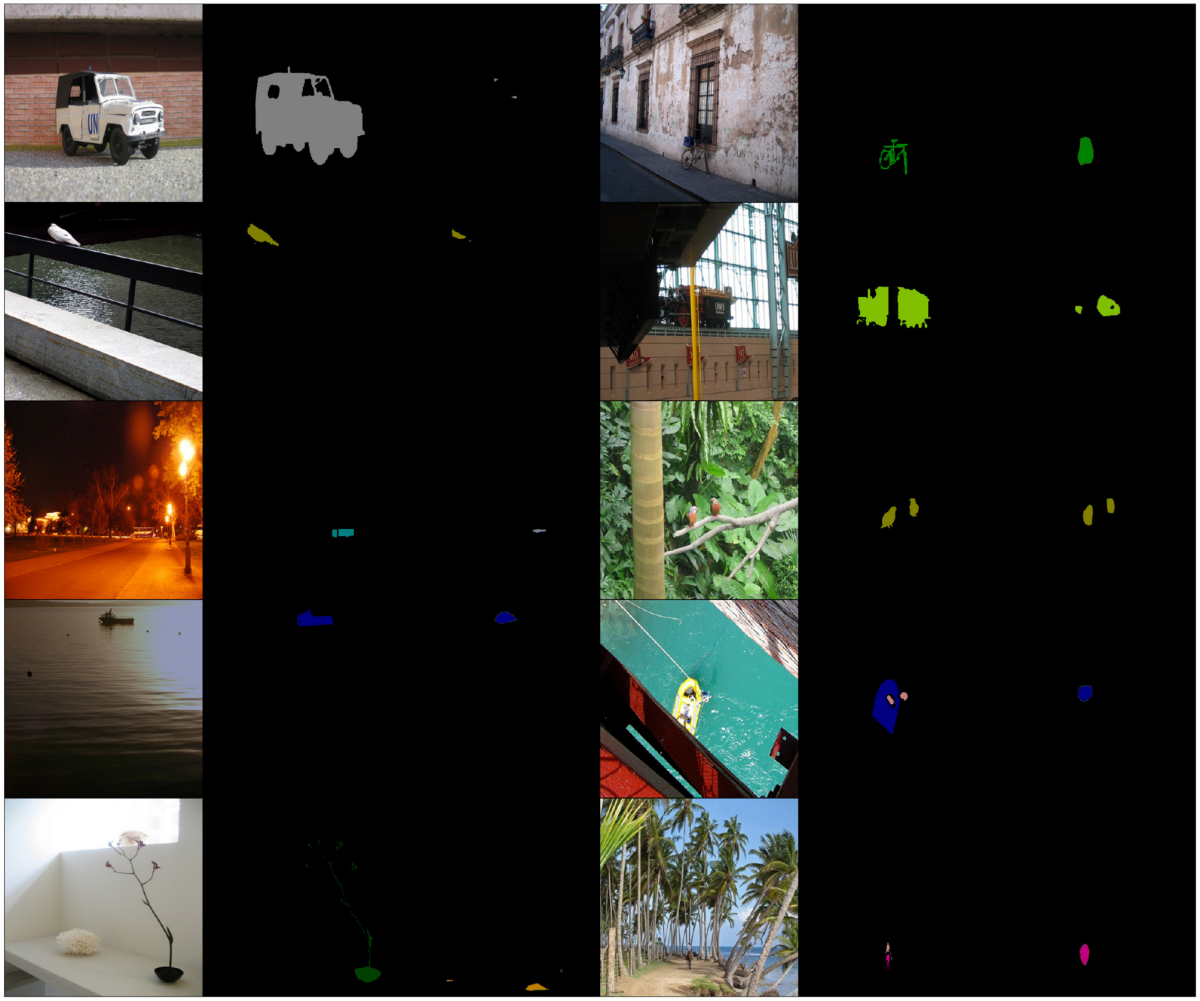
Random samples from Top-K = 50 GNSM rankings. Note how the predicted segmentations are either partial/missing or include incorrect classes. The columns (repeated twice) show input image, ground truth segmentations, and model predictions respectively. Different classes are denoted by color. The VOC data includes images obtained from Flickr: https://www.flickr.com/.

**Figure 3:**
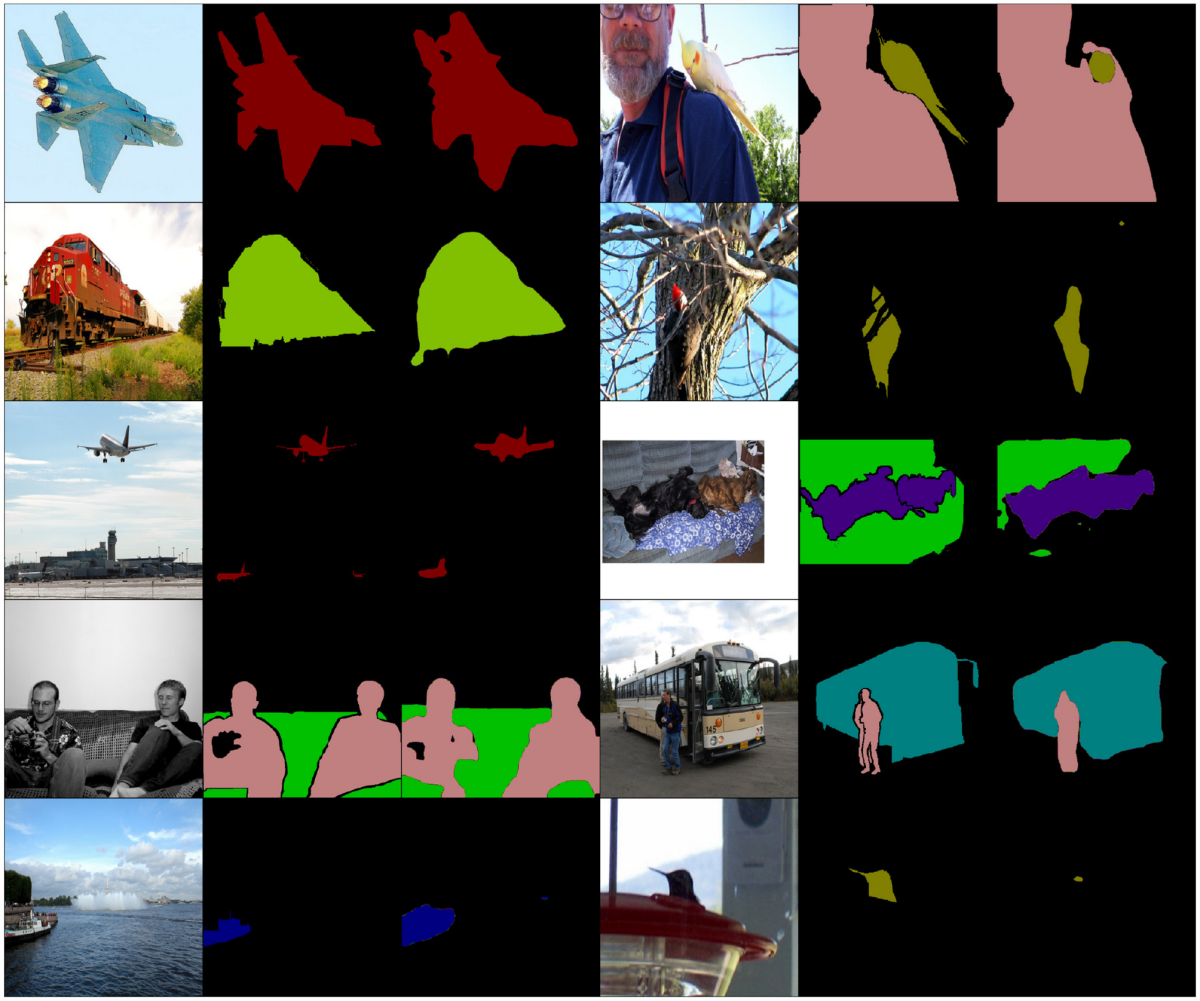
Random samples from Top-K = 50 DSVDD rankings. Note how only a few predictions may be considered anomalous. The VOC data includes images obtained from Flickr: https://www.flickr.com/.

To qualitatively assess the results of each model a subset of the worst ranked predictions are plotted in Figures 2, 3. We display examples of (image, ground-truth, DeepLab segmentation) triplets ranked as anomalous by GNSM and DSVDD, respectively. We expect the models to detect cases where the DeepLab model fails to produce adequate segmentations.

We observe that predictions ranked by GNSM in Figure 2 are either complete failures (most of the image is designated the background class) or severe under-segmentations. Predictions ranked by DSVDD in Figure 3 do not exhibit any obvious pattern of segmentation failures, with most being reasonable predictions. Our results show that, compared to DSVDD, GNSM is substantially more capable of detecting failure cases. Please look at the Appendix for all sorted *K* = 50 rankings.

We believe these results exemplify GNSM's generalization capabilities to non-tabular data, but also highlight a practical application. Quantifying segmentation uncertainties is useful when deploying off-the-shelf models. Our method may be employed as a filtering mechanism to automatically detect poor segmentations, which could then be reviewed further downstream.

## 7 Limitations

Our experiments revealed that GNSM's performance is closely tied to model architecture. While our proposed network size is performant, we observed a trend of increased performance as the models got deeper and wider. Due to time and resource constraints, we did not thoroughly explore the architecture space. This suggests that GNSM might benefit from larger models, which could be a limitation in resource-constrained environments.

Computationally, our models require a significant number of iterations to converge. For our experiments, we trained for 1 million iterations, which can take up to a day of training on an A6000 GPU. This is in contrast to the baselines, which may take a few seconds for shallow models and up to a few hours for the deep learning models.

Furthermore, GNSM explicitly needs to know the number of outcomes (classes) per category to appropriately add noise and compute the scores. While we believe this to be a strength of our approach, it does create an overhead for the user. The baselines do not require this additional modeling complexity and are more straightforward to apply.

Lastly, our method has hyperparameters pertaining to noise, such as the number of scales used and the range of noise levels. While our hyperparameters have proven to be stable across different datasets, we acknowledge that additional experiments for sensitivity would better illuminate the robustness of GNSM's hyperparameters. We posit that additional improvements may be obtained if these were also tuned per dataset.

## 8 Conclusion

In this work we introduced Gumbel Noise Score Matching (GNSM): a novel method for detecting anomalies in categorical data types. We outline how to compute scores of continuously relaxed categorical data and derive the appropriate training objective based on denoising score matching. Our method can easily be used in conjunction with standard score matching to model both continuous and categorical data. GNSM achieves competitive performance with respect to baselines on a suite of tabular anomaly detection datasets, attaining significant improvements on certain datasets. Furthermore, GNSM can easily be extended to images and excels on the real-world task of detecting anomalous segmentations. Lastly, we believe our novel categorical score matching formulation could be incorporated into generative models. We hope to explore this direction in future work.

## Data Availability

The tabular datasets analyzed for this study can be found at https://sites.google.com/site/gspangsite/sourcecode/categoricaldata. For the segmentation case study we used the PascalVOC Segmentation Dataset, retrieved from the Pytorch torchvision library at https://pytorch.org/vision/main/generated/torchvision.datasets.VOCDetection.html. Code for processing the datasets is available at https://github.com/ahsanMah/categorical-dsm/tree/.

## Footnotes

^1^ https://sites.google.com/site/gspangsite/sourcecode/categoricaldata

^2^ https://github.com/ahsanMah/categorical-dsm/tree/frontiers

^3^ https://pytorch.org/vision/stable//models/deeplabv3.html
