# Supplementary material for: Anomaly detection via Gumbel Noise Score Matching
Source: Front Artif Intell. 2024 Sep 24;7:1441205. doi: 10.3389/frai.2024.1441205 (PMC11488619; doi:10.3389/frai.2024.1441205)
Supplement: Supplementary file 1 [file Data_Sheet_1.pdf]

Supplementary Material

1 EXTENDED RESULTS

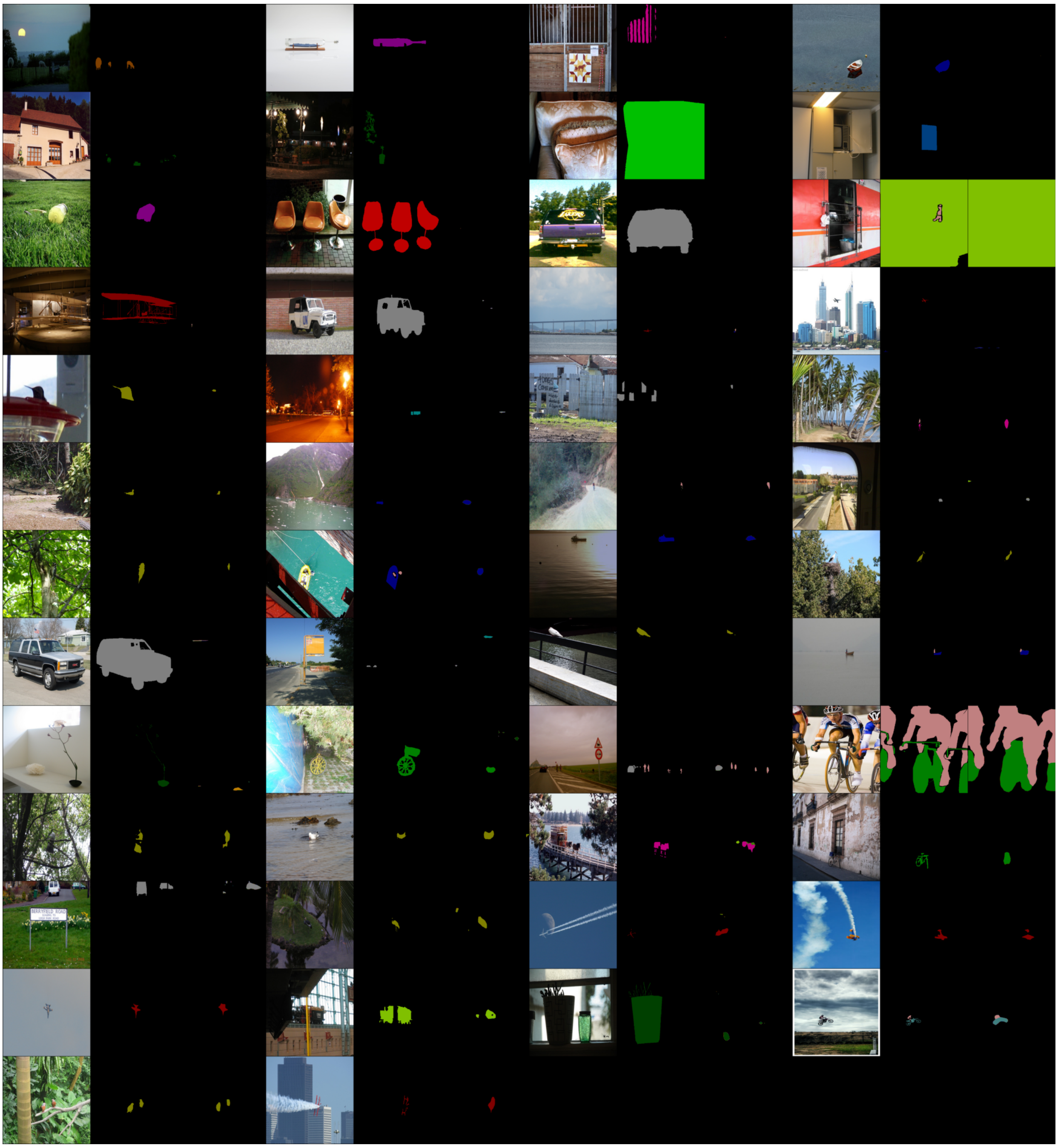

Figure S1. Samples from Top-K=50 GNSM rankings. The columns (repeated) show input image, ground truth segmentations, and model predictions respectively.

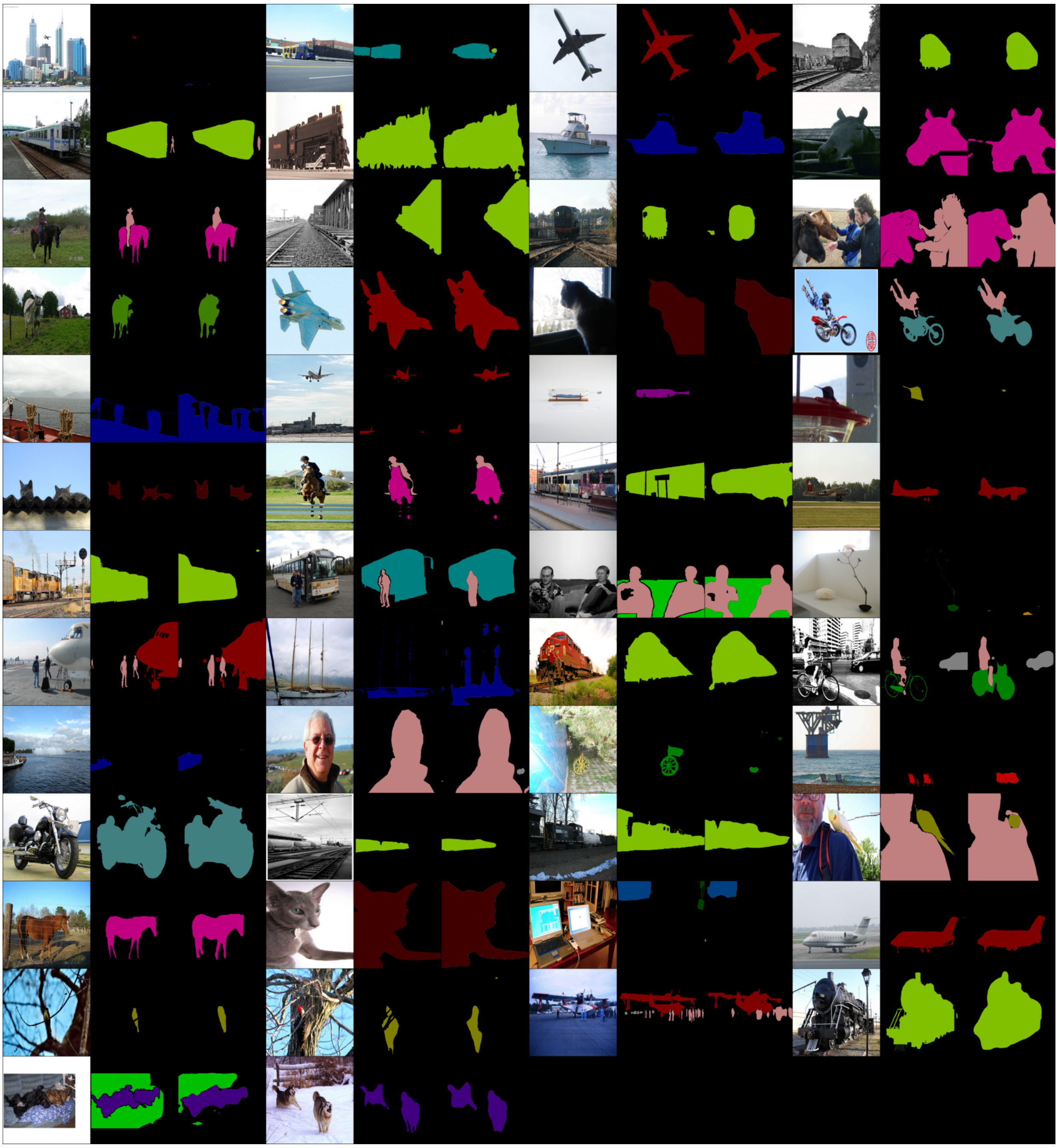

Figure S2. Random samples from Top-K=50 DSVDD rankings. The columns (repeated) show input image, ground truth segmentations, and model predictions respectively.

We show the predictions ranked poorly by both DSVDD and our method. Images are displayed in order from highest ranking to lowest (displayed left to right).

2 EXPERIMENT DETAILS AND HYPERPARAMETERS

2.1 GNSM Score Networks

Most of these details are easily identifiable in our open source code. However, we still provide basic information for posterity. We used the same ResNet-like architecture for all datasets:

$$\texttt{t} = \texttt{TimeEmbeddingLayer}(\lambda) \tag{S1}$$

$$\texttt{Net(x, t)} = \texttt{Head(ResBlock(... ResBlock(x, t)))} \tag{S2}$$

$$\texttt{ResBlock(x,t)} = \texttt{x + Linear(FiLM(x,t))} \tag{S3}$$

$$\texttt{Head} = \texttt{Linear(LeakyReLU(LayerNorm(x))} \tag{S4}$$

Note that the `FiLM` block is taken from (Perez et al., 2018) and the `TimeEmbeddingLayer` is the same as used in diffusion models (Song et al., 2021), using the `GaussianFourierProjection`. A simplified implementation of the ResBlock is shown below.

```
class TabResBlockpp(nn.Module):
    def __init__(self, d_in, d_out, time_emb_sz, act="gelu", dropout=0.0):

        self.norm = nn.LayerNorm(d_in)
        self.dense_1 = nn.Linear(d_in, d_out)
        self.act = get_act(act)
        self.film = FiLMBlock(time_emb_sz, d_out)
        self.dropout = nn.Dropout(dropout)
        self.dense_2 = nn.Linear(d_out, d_out)

    def forward(self, x, t):

        h = self.act(self.norm(x))
        h = self.dense_1(h)
        h = self.film(h, t)
        h = self.dropout(h)
        h = self.dense_2(h)

        return x + h
```

For Bank we trained for 2MM iterations while for CMC and Solar, we trained for 600K iterations (as they were significantly smaller datasets). All the other models were trained for 1MM iters. We used the AdamW optimizer with default paramaters. The learning rate was set to $1e-3$ with a cosine decay to $1e-5$ spanning the number of iterations. We also use an Exponential Moving Average of the weights at a decay rate of 0.999.The base config is shown below.

```
def get_config():
    config = ml_collections.ConfigDict()
    # training
    config.training = training = ml_collections.ConfigDict()
    training.batch_size = 2048 # Except for CMC and Solar where it was 512
    training.n_steps = 1000001
    training.snapshot_freq = 10000 # Number of iterations for checkpointing

    # evaluation
    config.eval = evaluate = ml_collections.ConfigDict()
    evaluate.batch_size = 1024

    # data config holds information about the dataset
    # such as number of categories
    config.data = data = ml_collections.ConfigDict()

    # default model parameters
    config.model = model = ml_collections.ConfigDict()
    model.name = "tab-resnet"
    model.tau_min = 2.0
    model.tau_max = 20
    ### Only relevant for Census
    model.sigma_min = 1e-1
    model.sigma_max = 1.0
    #####
    model.num_scales = 20
    model.ndims = 1024
    model.time_embedding_size = 128
    model.layers = 20
    model.dropout = 0.0
    model.act = "gelu"
    model.embedding_type = "fourier"
    model.ema_rate = 0.999

    # optimization
    config.optim = optim = ml_collections.ConfigDict()
    optim.weight_decay = 1e-4
    optim.optimizer = "AdamW"
    optim.lr = 1e-3
    optim.beta1 = 0.9
    optim.beta2 = 0.999
    optim.grad_clip = 1.0
    optim.scheduler = "cosine"
```

Lastly for MSMA, we train a GMM on the combined train and validation set. We run a small grid search over number of components (3,5,7,9) and pick the one with best likelihood.

2.2 DSVDD

For Deep SVDD we used the implementation available in the PyOD library Zhao et al. (2019). Initial testing showed that the autoencoder variant of this model usually performed better. This version adds a reconstruction loss to the one-class objective for increased regularization. The hidden neurons were set to [1024, 512, 256], with the `swish` activation function. Training was done with the Adam optimizer at default hyperparamters, with learning rate set to 1e-3. We trained for 1000 epochs, with the batch size set to 512.

2.3 DSVDD

For Deep SVDD we used the implementation available in the PyOD library Zhao et al. (2019). Initial testing showed that the autoencoder variant of this model usually performed better. This version adds a reconstruction loss to the one-class objective for increased regularization. The hidden neurons were set to [1024, 512, 256], with the `swish` activation function. Training was done with the Adam optimizer at default hyperparamters, with learning rate set to 1e-3. We trained for 1000 epochs, with the batch size set to 512.

2.4 DAGMM

DAGMM proved to be very difficult to train as most implementations often unexpectedly result in NaNs. In fact the implementation used by Han et al. (2022) never seemed to converge for any dataset. and the loss would not improve no matter how much we tweaked the hyperparameters. We believe the matrix inverse operation during the forward pass to be the culprit for this numerical instability.

We settled on modifiying a publicly available PyTorch implementation[1]. We added the following changes to improve stability and performance:

- Added Layer Normalization
- Added weight initialization
- Included checkpointing and early stopping using val set
- GMM parameters converted to double (float64)

Furthermore, we hand tuned hyperparameters for each dataset to find the most optimal (stable + performant) setting. Essentially, we tried to start from the same hyperparamters as DSVDD and tweaked until we got a stable model. We also early stopped on the checkpint that gave the best validation loss (tested every epoch). If a NaN was encountered before the first epoch was finished (i.e. before any checkpoint could be saved), we would restart training. The following hyperparameters were used for the final experiments:

```
hyp = {
    "input_dim": input_size,
    "hidden1_dim": 1024,
    "hidden2_dim": 512,
    "hidden3_dim": 256,
    "zc_dim": 2,
    "emb_dim": 128,
    "n_gmm": 2,
    "dropout": 0.5,
    "lambda1": 0.1,
```

[1] https://github.com/lixiangwang/DAGMM-pytorch

```
    "lambda2": 0.005,
    "lr": 1e-4,
    "batch_size": 256,
    "epochs": 1000,
    "patience_epochs": 10,
    "checkpoint": "best",
    "return_logits":False,
}

# Taken from KDDCUP-Rev config from original DAGMM paper
# Most other configs are unstable and frequently result in NaNs during training
if config.data.dataset in ["probe", "u2r"]:
    hyp["hidden1_dim"] = 120
    hyp["hidden2_dim"] = 60
    hyp["hidden3_dim"] = 30
    hyp["emb_dim"] = 10
    hyp["n_gmm"] = 4
    hyp["zc_dim"] = 1
    hyp["batch_size"] = 1024
    hyp["return_logits"] = True
    hyp["lr"] = 1e-5

if config.data.dataset == "bank":
    hyp["hidden1_dim"] = 64
    hyp["hidden2_dim"] = 32
    hyp["hidden3_dim"] = 16
    hyp["emb_dim"] = 10
    # hyp["zc_dim"] = 1
    hyp["batch_size"] = 4096
    hyp["lr"] = 1e-5

if config.data.dataset == "census":
    hyp["hidden1_dim"] = 256
    hyp["hidden2_dim"] = 128
    hyp["hidden3_dim"] = 64
    hyp["emb_dim"] = 10
    hyp["lr"] = 1e-5
```

2.5 ECOD and Isolation Forests

ECOD is hyperparamter free so no tuning was required. Early testing showed that Isolation Forests hyperparameters were stable. Note that we did not use labelled anomalies during hyperparamater tuning and the rest of the deep learning models were tuned on an inlier-only validation set.

REFERENCES

Han, S., Hu, X., Huang, H., Jiang, M., and Zhao, Y. (2022). ADBench: Anomaly detection benchmark. In *Thirty-sixth Conference on Neural Information Processing Systems Datasets and Benchmarks Track*

Perez, E., Strub, F., De Vries, H., Dumoulin, V., and Courville, A. (2018). Film: Visual reasoning with a general conditioning layer. In *Proceedings of the AAAI Conference on Artificial Intelligence*. vol. 32

Song, Y., Sohl-Dickstein, J., Kingma, D. P., Kumar, A., Ermon, S., and Poole, B. (2021). Score-based generative modeling through stochastic differential equations. In *International Conference on Learning Representations*

Zhao, Y., Nasrullah, Z., and Li, Z. (2019). Pyod: A python toolbox for scalable outlier detection. *Journal of Machine Learning Research* 20, 1–7
